# Mitochondrial DNA plasticity is an essential inducer of tumorigenesis

**DOI:** 10.1038/cddiscovery.2016.16

**Published:** 2016-04-04

**Authors:** W T Y Lee, J E Cain, A Cuddihy, J Johnson, A Dickinson, K-Y Yeung, B Kumar, T G Johns, D N Watkins, A Spencer, J C St John

**Affiliations:** 1 Centre for Genetic Diseases, Hudson Institute of Medical Research, Clayton, Victoria, Australia; 2 Department of Molecular and Translational Science, Monash University, Clayton, Victoria, Australia; 3 Centre for Cancer Research, Hudson Institute of Medical Research, Clayton, Victoria, Australia; 4 Myeloma Research Group, Division of Blood Cancers, Australian Centre for Blood Diseases, Monash University, Victoria, Australia; 5 Department of Pathology, Monash Health, Clayton, Victoria, Australia; 6 The Kinghorn Cancer Centre, Garvan Institute of Medical Research, Darlinghurst, New South Wales, Australia; 7 Faculty of Medicine, University of New South Wales, Randwick, New South Wales, Australia; 8 Department of Thoracic Medicine, St Vincent’s Hospital, Darlinghurst, New South Wales, Australia; 9 Malignant Haematology & Stem Cell Transplantation, Alfred Health, 55 Commercial Road, Melbourne, Victoria, Australia

## Abstract

Although mitochondrial DNA has been implicated in diseases such as cancer, its role remains to be defined. Using three models of tumorigenesis, namely glioblastoma multiforme, multiple myeloma and osteosarcoma, we show that mitochondrial DNA plays defining roles at early and late tumour progression. Specifically, tumour cells partially or completely depleted of mitochondrial DNA either restored their mitochondrial DNA content or actively recruited mitochondrial DNA, which affected the rate of tumorigenesis. Nevertheless, non-depleted tumour cells modulated mitochondrial DNA copy number at early and late progression in a mitochondrial DNA genotype-specific manner. In glioblastoma multiforme and osteosarcoma, this was coupled with loss and gain of mitochondrial DNA variants. Changes in mitochondrial DNA genotype affected tumour morphology and gene expression patterns at early and late progression. Importantly, this identified a subset of genes that are essential to early progression. Consequently, mitochondrial DNA and commonly expressed early tumour-specific genes provide novel targets against tumorigenesis.

## Introduction

The human mitochondrial genome (mtDNA) is 16.6 kb in size, circular and encodes 13 key genes of the electron transfer chain (ETC), which generates the vast majority of cellular ATP through the process of oxidative phosphorylation (OXPHOS).^1^ It also encodes 22 tRNAs and 2 RNAs and has one non-coding region, the D-loop. Disruption of the ETC due to mutation, deletion or depletion of mtDNA is associated with an increasing number of diseases.^2^ Although mtDNA copy number and variants have been associated with cancer,^3,4^ it remains to be determined whether the regulation of mtDNA copy number and the ability of mtDNA to acquire *de novo* variants are instrumental in driving tumorigenesis and, if so, how they affect the tumour phenotype. To test this, we have used three independent experimental models consisting of glioblastoma multiforme (GBM), a primary brain tumour that is neural in origin and a solid tumour;^5^ multiple myeloma (MM), a haematological tumour that originates from terminally differentiated B cells;^6^ and osteosarcoma, a solid tumour originating from osteoblast precursors.^7^ We show that mtDNA is essential for driving tumorigenesis and that complete mtDNA depletion prevents the onset of tumorigenesis. Furthermore, mtDNA copy number and variants are modulated at different stages of tumorigenesis. Altering a tumour cell’s mtDNA content results in changes to nuclear gene expression that directly affect the severity of the tumour phenotype.

## Results

### Modulating mtDNA copy number in MM cells disrupts tumorigenesis

**Figure 1:**
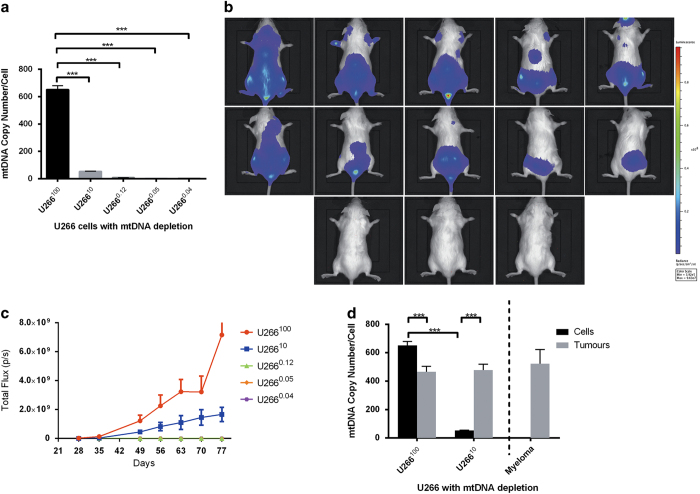
Undepleted and depleted U266 cells and tumour progression. (**a**) mtDNA copy number of U266 cells depleted to 10, 0.12, 0.05 and 0.04% of their original content; (**b**) *in vivo* tumour progression at day 49 (top row: U266^100^ tumours, second row: U266^10^, third row from left: U266^0.12^, U266^0.05^ and U266^0.04^); (**c**) tumour progression curve; and (**d**) mtDNA copy number in cells and tumours from U266^100^ and U266^10^ lines (***P<0.001).

To determine whether haematological tumours are dependent on mtDNA for tumorigenesis, we depleted human MM U266 cells labelled with luciferase to 10% (U266^10^), 0.12% (U266^0.12^), 0.05% (U266^0.05^) and 0.04% (U266^0.04^) of their original mtDNA content (U266^100^; Figure 1a) and transplanted them and non-depleted (U266^100^) cells into immunocompromised mice. After 35 days, each inoculation of U266^100^ cells developed into tumours (Figure 1b) and, by 77 days, had spread to the hindlimbs causing paralysis, as anticipated.^8^ Although U266^10^ cells produced tumours in each mouse by 42 days (Figure 1b), tumours grew at a significantly slower rate (Figure 1c) and their localization was less focal. At 84 days, only 20% of the mice developezd hindlimb paralysis. Nevertheless, U266^10^ tumours recovered mtDNA copy number to levels similar to U266^100^ tumours (Figure 1d). However, tumours did not arise from U266^0.12^, U266^0.05^ and U266^0.04^ cells (Figures 1b and c). These results, along with the previous findings in our GBM tumour model,^9^ suggest that a cell-specific threshold of mtDNA copy number is essential for the propagation and growth of tumours in solid and now blood cancers.

### Tumour cells unable to replenish mtDNA recruit mtDNA

**Figure 2:**
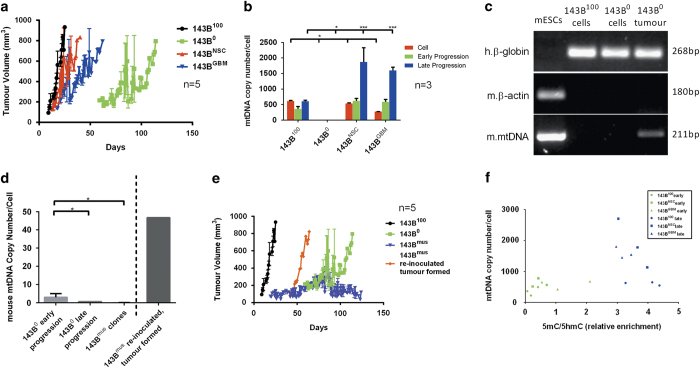
143B cells and 143B cells harbouring different mtDNA genotypes. (**a**) Tumour progression for 143B^100^, 143B^0^, 143B^NSC^ and 143B^GBM^ cells; (**b**) mtDNA copy number in 143B^100^, 143B^0^, 143B^NSC^ and 143B^GBM^ cells, and their respective tumours at early and late progression; (**c**) PCR-gel of mouse mtDNA in 143B^0^ tumours. Human (h.)*β*-globin and mouse (m.)*β*-actin demonstrate the presence of human and mouse chromosomal DNA, respectively, while m.mtDNA shows the presence of mouse mtDNA; (**d**) mouse mtDNA in 143B^0^ tumours at early progression, late progression, cells isolated from late progression tumours and after further cultured *in vitro* (143B^mus^ clones), and tumours from re-inoculated 143B^mus^ cells; (**e**) tumour progression for isolated 143B^0^ tumour cells post-reinoculation (143^mus^ reinoculated tumour formed); (**f**) 143B^100^, 143B^GBM^ and 143B^NSC^ tumours at early and late progression underwent immunoprecipitation to assess the levels of enrichment for 5-methylcytosine (5mC) and 5-hydroxymethylcytosine (5hmC), which are indicative of active *de novo* and transient DNA methylation, respectively, and mtDNA copy number to determine the replicative efficiency (*P<0.05; ***P<0.001).

Since partially mtDNA-depleted tumour cells replenish their mtDNA to initiate tumorigenesis, we inoculated immunocompromised mice with human osteosarcoma cells, 143BTK−, that were completely depleted of and unable to replenish mtDNA (143B^0^).^10,11^ While their non-depleted (143B^100^) counterparts started to develop tumours 9 days post-inoculation and reached late progression (~800 mm^3^) by day 25, tumour development in 143B^0^ cells was initiated in only 60% of mice by day 59 and progressed at a much slower rate (Figure 2a). Although 143B^100^ tumours possessed mtDNA at preinoculation levels, 143B^0^ tumours were unable to recover their endogenous mtDNA (Figure 2b). However, 143B^0^ tumour cells, FACS-sorted to eliminate contaminating mouse stroma, possessed mouse mtDNA (143B^Mus^; Figure 2c) most likely acquired from the surrounding *in vivo* microenvironment.^12^ Colonies established from FACS-sorted single cells possessed ~1 copy per cell (Figure 2d; 143^Mus^ clones). However, as human nuclear mtDNA replication factors cannot recognize murine mtDNA promoters,^13^ mtDNA is recruited prior to early progression (~50 mm^3^) and is thus diluted out by late progression (Figure 2d). Consequently, 143B^Mus^ cells were re-inoculated into mice to determine whether cells already harbouring more genetically divergent mtDNA would support full tumour development and acquire additional mtDNA. All five mice developed tumours; however, only one proceeded to late progression and had accelerated growth (Figure 2e). The other four mice developed tumours to ~200–300 mm^3^, which then arrested and ultimately regressed. Furthermore, tumours from re-inoculated 143B^Mus^ cells possessed significantly higher levels of mtDNA (Figure 2d), again indicating that mtDNA is acquired to sustain tumorigenesis. Collectively, these results demonstrate that mtDNA is essential to the onset of tumorigenesis and mtDNA is acquired from the surrounding tumour environments when endogenous mtDNA is unavailable.

### MtDNA copy number varies among developing tumours with different mtDNA genotypes

To determine if tumour cells modulate mtDNA copy number based on their mtDNA genotype, we repopulated 143B^0^ cells with mtDNA from human neural stem cells (NSC) and HSR-GBM1 cells to generate 143B^NSC^ and 143B^GBM^ cell lines. The presence of NSC and HSR-GBM1 mtDNA was confirmed by sequencing of the D-loop regions of 143B^NSC^ and 143B^GBM^ cells (Supplementary Extended Data Figure S1). MtDNA copy number was higher in 143B^100^ and 143B^NSC^ cells than that of 143B^GBM^ cells (Figure 2b). To assess the tumorigenic potential of cells harbouring different mtDNA genotypes, mice were inoculated with 143B^NSC^ and 143B^GBM^ cells, which developed into tumours during the first 20 days. Tumours derived from 143B^NSC^ cells had marginally slower growth rates than from 143B^100^ cells, while tumours from 143B^GBM^ cells grew more slowly than 143B^100^ and 143B^NSC^ cells (Figure 2a). During tumorigenesis, mtDNA copy number was modulated according to the stage of development. 143B^100^ cells reduced mtDNA copy number at early progression but reverted to preinoculated levels by late progression (Figure 2b), similar to MM tumours (Figure 1d) derived from depleted cells. However, 143B^NSC^ and 143B^GBM^ tumours had significantly higher levels of mtDNA copy number at early and late progression (Figure 2b; *P*<0.05). As mtDNA copy number is regulated in a tissue-specific manner by DNA methylation at exon 2 of the nuclear-encoded catalytic subunit of the mtDNA-specific Polymerase Gamma (*POLGA*),^14,15^ we assessed the mtDNA replicative efficiency in preinoculated cells and at early and late progression. As demonstrated by the distinct patterns of clustering for the three cohorts (Figure 2f), mtDNA copy number is differentially regulated at various stages of tumorigenesis, mediated by DNA methylation at exon 2 of POLGA. These outcomes emphasize the plasticity of mtDNA copy number and DNA methylation of POLGA at exon 2 to promote tumorigenesis with adaptation of mtDNA copy number dependent on genotype.

Haematoxylin and eosin staining showed distinctive phenotypes between each of the tumours harbouring different mtDNA genotypes. Similar to 143B^100^ tumours, 143B^NSC^ tumours were predominantly sarcomatoid in appearance but exhibited focal regions of polygonal cell morphology consistent with a carcinoma phenotype (Supplementary Extended Data Figures S2a and b), indicative of mesenchymal–epithelial transition (MET) and increased invasiveness.^16^ 143B^GBM^ tumours exhibited characteristics of differentiation and vascular invasion (Supplementary Extended Data Figures S2c and d). 143B^0^ tumours displayed morphology indicative of angiosarcoma (Supplementary Extended Data Figure S2e), while reinjected 143B^Mus^ tumours resembled high-grade lieomyosarcoma and rhobdomyosarcoma (Supplementary Extended Data Figure S2f). Consequently, mtDNA content induces phenotypic differences in tumours.

### Different regions of mtDNA are susceptible to variants in MM and HSR-GBM1 tumours

Since tumours harbour a range of mtDNA variants,^17^ we assessed mtDNA next generation sequences from tumours generated from our GBM and MM models to determine which regions of mtDNA were more susceptible to the accumulation of variants. While HSR-GBM1 cells were most susceptible to mtDNA variants in *COX2* (complex IV of the ETC) and the D-Loop for the coding and non-coding regions, respectively, tumours derived from HSR-GBM1 cells possessing either 100% mtDNA or depleted to varying levels^9^ gained variants as a result of tumour formation^18^ (Supplementary Extended Data Table S1). As a result, *ND6* (complex I) became the most susceptible coding region to variants. In similar fashion, *ATP8* and the D-Loop were the most susceptible to variants in U266^100^ cells (Supplementary Extended Data Table S2). However, U266^10^ cells and tumours derived from U266^100^ and U266^10^ cells resulted in *ND6* being the most susceptible gene region most likely arising from an overall loss of variants in the coding regions during tumorigenesis and the gain of three *de novo* variants including the variant at position 14505 (*ND6*). Consequently, *ND6* appears to be a highly susceptible region in tumours of solid and blood origin (Supplementary Extended Data Tables S1 and S2).

### Changes in susceptibility to mtDNA variants at early and late progression

To determine whether mtDNA variants were modulated during tumorigenesis, we assessed variants in our osteosarcoma model. For 143B^100^ cells, the number of variants decreased from 18 to 10 as they proceeded to early progression and increased to 15 at late progression (Supplementary Extended Data Table S3). There were five common variants at all stages, two in the D-Loop and three in the coding regions. A further four variants were present in the coding regions of cells and at late progression but not at early progression. Nevertheless, as for MM and GBM, *ND6* was the most susceptible to variants in cells and at late progression while *ATP8* was the most susceptible at early progression. Similar patterns of modulation were observed for our 143B^GBM^ and 143B^NSC^ models (Supplementary Extended Data Table S3). 143B^GBM^ cells harboured 24 variants. Six variants were common to cells and late progression, with *ATP8* being the most susceptible at these stages. Only 10 variants were identified in 143B^NSC^ cells; however, at early progression, this was reduced to 6 followed by an increase to 14 at late progression (Supplementary Extended Data Table S3). Eight variants were common to cells and late progression. As a result, *ND3* was the most susceptible to variants in cells and late progression while *COX2* was the most susceptible at early progression. This highlights the dynamic nature of the recruitment of mtDNA variants during tumorigenesis. However, changing mtDNA genotypes results in different mtDNA coding genes becoming more susceptible to variants.

### MtDNA copy number, variants and genotype modulate osteosarcoma gene expression

The 143B cell lines and tumours were analysed by a whole-genome expression microarray to determine how mtDNA genotypes influence chromosomal gene expression during tumorigenesis, especially as certain mtDNA haplotypes are predisposed to cancer.^19,20^ There were 951 genes differentially expressed between the 143B^100^ and 143B^0^ tumours at late progression. The three most affected networks were: (1) nervous system development and function, drug metabolism and molecular transport; (2) RNA post-transcriptional modification, infectious disease and haematological disease; (3) and cellular assembly and organization, cellular function and maintenance, and connective tissue development and function (Supplementary Table S1). Specifically, the hepatic fibrosis, RhoGDI signalling, mitochondrial function and angiogenesis pathways were significantly downregulated in 143B^0^ tumours (Supplementary Extended Data Table S4). This was supported by upregulation of NFKB1A and NFKB1Z, which inhibit NF-*κ*B activity and suppress proangiogenic molecules such as MMP9,^21,22^ and are associated with osteosarcoma.^23^ Likewise, other immune-related genes, *CXCL8, IL4R* and *IL10*, were upregulated to promote cytokine responses and suppress tumorigenesis. Furthermore, genes regulating insulin-like growth factor action and bone formation were downregulated in 143B^0^ tumours.

By comparing 143B^GBM^ and 143B^NSC^ with 143B^0^ at late progression, we identified 379 differentially expressed genes. The three most affected networks were: (1) carbohydrate metabolism, amino acid metabolism and post-translational modification; (2) cellular development, haematological system development and function and hematopoiesis; (3) and connective tissue disorders, cardiovascular system development and function, and cellular development (Supplementary Table S2). Again, the majority of genes affected were associated with hepatic fibrosis and RhoGDI signalling (Supplementary Extended Data Table S4). Genes upregulated in the 143B^NSC^ and 143B^GBM^ tumours included those involved in glycolysis, highlighting the importance of this metabolic pathway to tumorigenesis. Among the genes involved in the hepatic fibrosis pathway, expression of the *IGFBP*s*, COL4A1, COL4A2*, *MMP9* and *TNFRSF11B* was restored in 143B^NSC^ and 143B^GBM^ tumours. Furthermore, Cadherin-11 (CDH11), a marker of MET in osteosarcoma,^24^ was upregulated in 143B^GBM^ and 143B^NSC^ tumours, indicating partial differentiation and metastasis. These outcomes indicate that the introduction of mtDNA activates key gene pathways to promote tumorigenesis.

**Figure 3:**
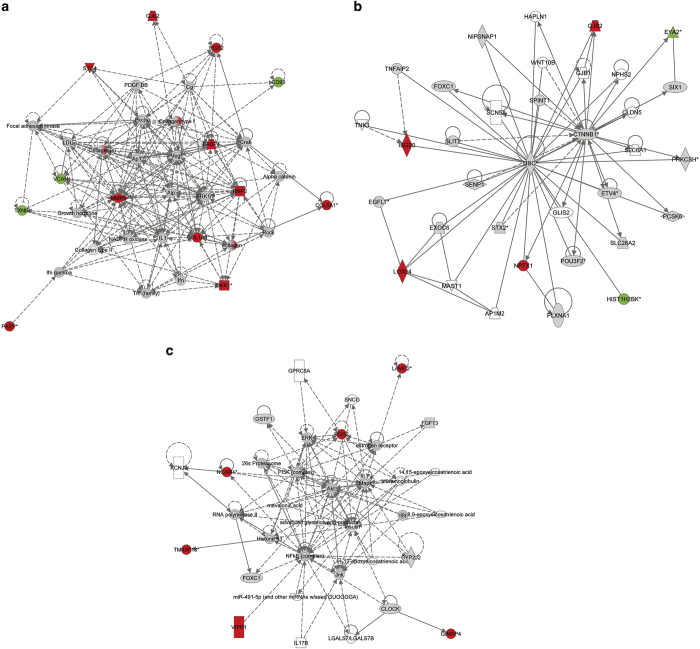
Figure 3. Network analysis based on differentially expressed genes between 143BNSC and 143BGBM tumours in (**a**) cancer, cardiovascular system development and function, and organismal development, (**b**) cancer, gastrointestinal disease and hepatic system disease, and (**c**) cell cycle, cardiovascular system development and function networks (red – upregulated in 143BNSC; green – downregulated in 143BNSC, fold change>2, *P*<0.05).

We identified 24 differentially expressed genes between the 143B^NSC^ and 143B^GBM^ tumours. The affected networks were (1) cancer (Figures 3a and b; *P*<0.05), cardiovascular system development and function, and organismal development; (2) cell cycle (Figure 3c), cardiovascular system development and function, and cellular movement; (3) and cancer, gastrointestinal disease and hepatic system disease (Supplementary Extended Data Table S5). Neural lineage markers, such as *NCAM1* (early) and *NPTX1* (neuronal), were highly expressed in 143B^NSC^ tumours, whereas genes involved in cancer proliferation, including *EYA2, VCAN, HMGA1* and *TXNIP*, were highly expressed in 143B^GBM^ tumours. The affected pathways were associated with adipogenesis, hepatic fibrosis and osteoblast formation (Supplementary Table S3). This highlights the cell-specific induction of nuclear gene expression at late progression by different mtDNA genotypes.

**Figure 4:**
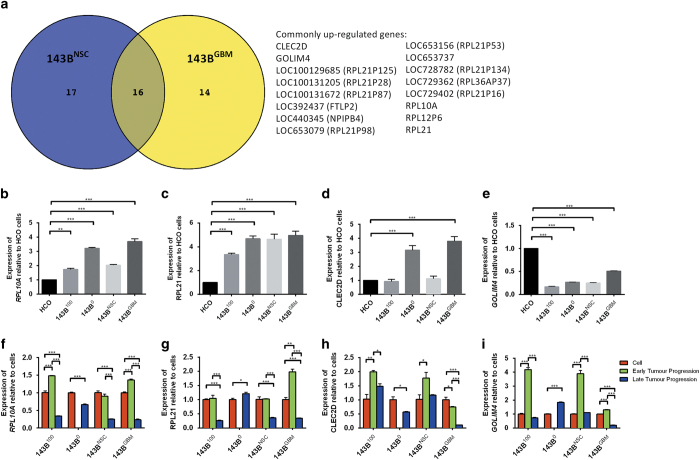
Differential gene expression. (**a**) Sixteen common genes that were upregulated at early tumour progression but downregulated at late progression between 143B^NSC^ and 143B^GBM^ tumours; (**b**) expression of RPL10A in all 143B cell lines relative to HCO cells; (**c**) expression of RPL21 in all 143B cell lines relative to HCO cells; (**d**) expression of CLEC2D in all 143B cell lines relative to HCO cells; (**e**) expression of GOLIM4 in all 143B cell lines relative to HCO cells; (**f**) expression of RPL10A in all 143B cells and at early and late progression; (**g**) expression of RPL21 in all 143B cells and at early and late progression; (**h**) expression of CLEC2D in all 143B cells and at early and late progression; and (**i**) expression of GOLIM4 in all 143B cells, and at early and late progression (*P<0.05; **P<0.01; ***P<0.001).

To determine whether gene expression patterns were differentially regulated by mtDNA genotypes at different stages of tumour development, we analysed gene expression patterns in 143B^GBM^ and 143B^NSC^ tumours at early and late progression. We identified 33 differentially expressed genes between early and late 143B^NSC^ tumours (Supplementary Table S4) and 30 for the 143B^GBM^ tumours (Supplementary Table S5). Furthermore, 16 genes were upregulated in both the early 143B^GBM^ and 143B^NSC^ tumours and downregulated at late progression (Figure 4a; *P*<0.05, *q*<0.05). Among the common genes, there were 4 functional genes, namely *RPL21, RPL10A, CLEC2D* and *GOLIM4*; and 10 pseudogenes, 7 for *RPL21*, and 1 each for *RPL12, RPL36A* and *FTL*.

Since *RPL21* and *RPL10A* are highly expressed in cancer,^25,26^ we confirmed their upregulation in our model of tumorigenesis. *RPL10A* and *RPL21* were more highly expressed in the 143B^100^, 143B^GBM^ and 143B^NSC^ cell lines than human calvarial osteoblasts (HCO) (Figures 4b and c; *P*<0.01). There was no significant difference for *RPL21* expression amongst 143B^100^, 143B^GBM^ and 143B^NSC^ cells and early progression, but expression was significantly higher at early than at late progression (Figure 4g; *P*<0.05). *RPL10A* expression was significantly elevated during early 143B^100^, 143B^NSC^ and 143B^GBM^ progression (Figure 4f; *P*<0.001). Expression of *CLEC2D*, which encodes lectin-like transcript 1 (LLT1) and blocks osteoclast differentiation,^27^ was only significantly increased in the 143B^0^ and 143B^GBM^ cells when compared with HCO (Figure 4d; *P*<0.001). However, expression of *CLEC2D* was significantly upregulated at early progression compared with late progression (Figure 4h; *P*<0.05). Expression of *GOLIM4*, a Golgi integral membrane protein, was lower in tumour cells compared with HCO (Figure 4e; *P*<0.001), and upregulated in 143B^100^, 143B^NSC^ and 143B^GBM^ tumours at early progression (Figure 4i; *P*<0.001) and downregulated at late progression (Figure 4i; *P*<0.001). Therefore, by modulating mtDNA genotypes in tumour cells, we have identified a common core of essential genes specific to early progression.

### Modulating mtDNA copy in MM cells affects tumorigenic gene expression

**Figure 5:**
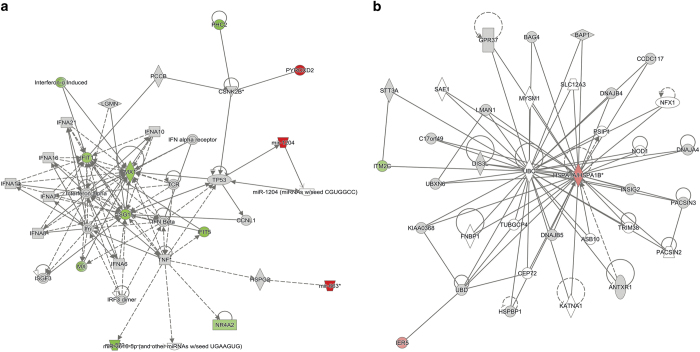
Differentially expressed genes between U266^100^ and U266^10^ in (**a**) cells and (**b**) tumours. (red – upregulated in U266^10^; green – downregulated in U266^10^, fold change >2, *P*<0.05).

To determine whether modulation of mtDNA copy number in tumour cells induces changes to gene expression, we performed RNA-seq on U266^100^ and U266^10^ cells and tumours. Between U266^100^ and U266^10^ cells, 16 genes were differentially expressed primarily affecting the cellular development, haematological system development and function, and hematopoiesis; connective tissue disorders; and developmental disorder networks (Supplementary Table S6). The canonical interferon signalling pathway was most affected, with type I interferon-related genes *IFIT1, MX1, IFIT5* and *ISG15* significantly downregulated in U266^10^ cells along with the prosurvival gene *NR4A2*, which suppresses the assembly of tumour suppressor P53^28,29^ (Figure 5a; *P*<0.05). Furthermore, *MIR-663* was upregulated in U266^10^ cells (Figure 5a). MIR-663 targets TGFB1 and regulates the expression of *PTEN*, a tumour suppressor gene^30^ and downregulates BCL-2 to induce apoptosis.^31^ Consequently, in U266 cells, mtDNA depletion significantly decreases the expression of interferon-related genes, reduces P53 suppressor activity and activates tumour-suppressing micro-RNAs, which alter the tumorigenic state of U266 cells. However, in U266^10^ tumours, gene expression was restored as a consequence of replenishing mtDNA, as only a few differentially regulated genes were identified that affected the post-translational modification, protein folding, cancer, cellular assembly and organization networks (Supplementary Extended Data Table S6). Primarily, *HSPA1A, HSPA1B* and *ITM2C* were upregulated (Figure 5b; *P*<0.05), which alter the expression of heat-shock protein (Hsp) 70 and prevent TNF-induced apoptosis.^32–34^ These subtle changes likely account for the lack of specificity exhibited by U266^10^ cells, for example, their failure to induce paralysis.

### Modulating mtDNA copy in GBM cells affects tumorigenic gene expression

**Figure 6:**
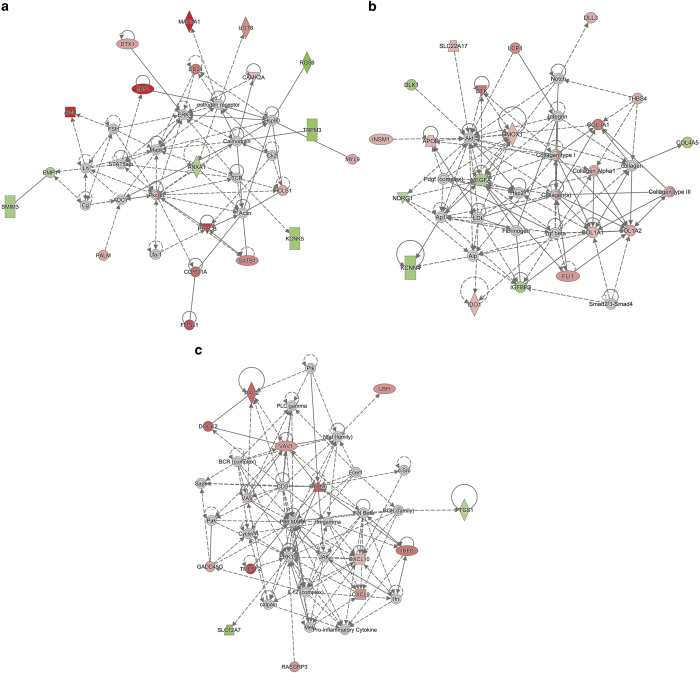
Differentially expressed genes between GBM^100^ and GBM^3^ and GBM^0.2^ tumours involved in (**a**) cellular development, (**b**) cancer and (**c**) cellular function and maintenance networks (red – upregulated in GBM^3^ and GBM^0.2^; green – downregulated in GBM^3^ and GBM^0.2^, fold change >2, *P*<0.05).

To determine whether the changes in gene expression resulting from the replenishment of mtDNA copy number to promote tumorigenesis were more widespread, we analysed our GBM tumour model. There were 101 differentially expressed genes between GBM^0.2^ and GBM^3^ tumours and GBM^100^ tumours with the most affected networks being: (1) cellular development, cellular growth and proliferation, and haematological system development and function; (2) cancer, gastrointestinal disease, and organismal injury and abnormalities; (3) and cancer, organismal injury and abnormalities, and reproductive system disease (Supplementary Table S7). The increased expression of genes associated with B-cell receptor signalling and the cytoskeleton likely promoted cell proliferation and tumour formation,^35–39^ thus compensating for the loss of tumorigenicity in GBM^0.2^ and GBM^3^ cells^9^ (Supplementary Extended Data Table S7 and Figures 6a–c; *P*<0.05).

Comparison of the more aggressive GBM^50^ tumours and GBM^100^ tumours identified 136 differentially expressed genes. The cancer, cell cycle, cellular development; skeletal and muscular system development and function, skeletal and muscular disorders, cardiovascular system development and function; and connective tissue disorders, cellular assembly and organization, cellular function and maintenance were the most affected networks (Supplementary Table S8). Of the affected genes, 12 were cancer specific (Supplementary Extended Data Figure S3; *P*<0.05), which included a key regulator of autophagy, hypoxia-related genes and a modulator of the Wnt/*β*-catenin signalling pathway. Collectively, these changes in gene expression promote migration and invasion of tumour cells.^40–42^

## Discussion

Our data demonstrate that mtDNA is an essential driver of tumorigenesis. Indeed, a tumour cell’s mtDNA content undergoes dynamic changes during tumorigenesis, which likely reflects changes in the aerobic nature of the microenvironment and the cell’s requirement for ATP derived from OXPHOS. In our three models, copy number is modulated at early and late progression and, in GBM and osteosarcoma, this is matched by changes in regions of the genome susceptible to variants. The Complex I gene, *ND6*, is the most affected, which is the only protein coding gene on the light strand and is the last of these genes to be replicated,^43^ and is thus most prone to the proofreading error that affects *POLGA*.^44^ As ND6 is essential to assembly and function of Complex I, its dysfunction would therefore promote a shift to glycolysis for ATP generation.^45^ In addition, hypermethylation of *POLGA* limits the numbers of mtDNA copy available for transcription, thus further promoting aerobic glycolysis. Nevertheless, cells lacking mtDNA have the potential to initiate tumorigenesis by acquiring it from the surrounding stroma. However, 143B^0^ cells that acquire mouse mtDNA and U266 and GBM cells possessing lower levels of mtDNA are less tumorigenic while those completely depleted cells that do not acquire mtDNA are non-tumorigenic and are thus unable to modulate mtDNA to drive tumorigenesis.

Remarkably, in all three models, a tumour cell’s mtDNA copy number and genotype can determine the rate of onset for tumorigenesis, morphology and gene expression profiles. Specifically, in our osteosarcoma model, we show that a critical set of genes is present at early progression. While mtDNA haplotypes have been associated with a host of phenotypes and diseases,^46^ it is evident that a subset of key genes is recruited at early progression. Other genes, in line with mtDNA copy number and specific to haplotype, would be recruited to promote transition to late progression in order to define the tumours end point phenotype. Consequently, targeting mtDNA and commonly expressed early tumour-specific genes would provide novel candidate approaches against tumour formation.

## Materials and Methods

### Mice

NOD.Cg-*Prkdc*^*scid*^ *Il2rg*^*tm1Wjl*^/SzJ (NSG) mice were obtained from Jackson Laboratories (Bar Harbor, Maine, USA) and kept in SPF facilities. Animal work using MM cells was approved by the Alfred Medical Research and Education Precinct (AMREP) Animal Ethics Committee (Ethics number E/1364/2013/M). Female BALB/c Nude mice were obtained from the Animal Resources Centre, Australia. Experiments using 143B cell and animals were approved by Animal Ethics Committee at Monash University (Ethics number MMCA 2012/24). All mouse work was carried out in accordance with the ‘Australian Code of Practice for the Care and Use of Animals for Scientific Purposes.*’*

### Cell lines and viral transduction

The human myeloma cell line U266 was obtained from ATCC (Manassas, VA, USA) and maintained in single-cell suspensions in RPMI 1640 media supplemented with 10% fetal bovine serum (FBS), 2 mM GlutaMax and 1 mM sodium pyruvate (all from Gibco Life Technologies, Carlsbad, CA, USA). U266 cells suitable for bioluminescent imaging were transduced by spinfection in the presence of 4 *μ*g/ml polybrene and supernatant containing lentiviral particles with the FUL2-TG vector kindly provided by Dr. Marco Herold (Walter and Eliza Hall Institute, Melbourne, Australia). The FUL2-TG vector contains luciferase-2 and GFP driven by the ubiquitin promoter.^47^ GFP+ cells were sterile sorted prior to transplant using an Influx cell sorter (Becton Dickinson, North Ryde, NSW, Australia).

### Culture of multiple myeloma cell lines

U266 cells were cultured in single-cell suspensions in RPMI 1640 media supplemented with 10% FBS, 2 mM GlutaMax and 1 mM sodium pyruvate (all from Life Technologies, Carlsbad, CA, USA). MtDNA depletion was performed by the addition of 10*μ*g/ml of 2′,3′-dideoxycytidine (ddC), in the presence of 50*μ*g/ml of uridine (both from Sigma, St Louis, MO, USA). For the recovery of mtDNA, cells were cultured in complete RPMI media in the absence of ddC, or in the presence of the glycolysis inhibitor, 2-deoxyglucose (2-DG), at a concentration of 5 mM

### Culture of osteosarcoma (143BTK−) cell lines

Human osteosarcoma (143B) cells, which were deficient in thymidine kinase activity (143BTK−) and 143B^0^ cells, which were previously completely depleted of mtDNA by long-term exposure of ethidium bromide,^10,11^ were kindly donated by Assoc Prof. Ian Trounce, Centre for Eye Research, Australia. All 143BTK− cell lines were routinely cultured in standard DMEM (SD-DMEM) consisting of DMEM, 10% (v/v) FBS, 10 mM sodium pyruvate, 2 mM GlutaMax and 1% (v/v) penicillin/streptomycin (Life Technologies). 143B^0^ cells were supplemented with uridine (Sigma-Aldrich, Sydney, NSW, Australia) at a final concentration of 50 *μ*g/ml. All cell lines were cultured at 37 °C in 5% CO_2_ and 95% humidity.

### Generation of 143B^NSC^ and 143B^GBM^ cells

143B^0^ cells were repopulated with donor mtDNA through fusion with cytoplasts from human neural stem cells (hNSC) derived from the embryonic stem cell line WA09 and human glioblastoma multiforme cells (HSR-GBM1) to generate trans-mitochondrial cybrids, according to methods previously described.^48^

In brief, 3×10^6^ hNSC, HSR-GBM1 cells were each resuspended in 10 ml of SD-DMEM and mixed with 10 ml of Percoll solution (Sigma) pre-warmed at 37 °C, 200 *μ*l penicillin/streptomycin and 2 mg/ml cytochalasin B (Sigma) in 50 ml Nalgene high-speed centrifuge tubes (Nalgene, Waltham, MA, USA). Cells were centrifuged at 20 000 r.p.m. using a SS-34 fixed angle rotor for 70 min at 27 °C to enucleate. Percoll/media interface (7.5 ml) was transferred to 15 ml tubes and 7.5 ml of fresh SD-DMEM was added to the cell suspension and centrifuged at 4400 r.p.m. for 5 min. Following centrifugation, the supernatant was removed and the cell pellets were resuspended in 10 ml of SD-DMEM.

1×10^6^ 143B^0^ cells were mixed with 10 ml cell suspension of hNSCs or HSR-GBM1 cells in 50 ml Nalgene high-speed centrifuge tubes and centrifuged at 10 000 r.p.m. using the SS-34 fixed angle rotor at 27 °C for 10 min. Cell pellets were collected and covered with 500 *μ*l of cell culture grade polyethylene glycol (PEG; Sigma) for 1 min. PEG was immediately removed and the cell pellets were resuspended in 10 ml of SD-DMEM to complete fusion.

The fusion mixtures were then cultured in SD-DMEM for 24 h at 37 °C in 5% CO_2_ and 95% humidity and transferred to the selection media, CSM, consisting of RPMI medium (Life Technologies), 5% FBS, 2 mM GlutaMax, 1% penicillin/streptomycin and 5-bromo-2-deoxyuridine (BrdUrd; Sigma) at a final concentration of 20 *μ*g/ml. The medium was replaced every 2 days. After 7–14 days, propagated cell colonies were isolated and expanded in CSM media. The fused cells containing mtDNA from hNSCs and HSR-GBM1 were named as 143B^NSC^ and 143B^GBM^, respectively.

### MtDNA genotyping

MtDNA genotyping was subsequently performed using capillary sequencing to confirm transfer of desired mtDNA populations into the recipient 143B^0^ cells. Purified DNA from control 143BTK− (143B^100^), 143B^0^, 143B^NSC^ and 143B^GBM^ was amplified using conventional PCR with primers that amplify the human D-Loop region (Table 1). PCR products were confirmed on 2% agarose gels (Bioline, Sydney, NSW, Australia) and the desired bands were excised and purified using the QIAquick Gel Extraction Kit (Qiagen, Valencia, CA, USA). Samples were sequenced through the Monash Health Translational Precinct (MHTP) Medical Genomics Facility (Hudson Institute of Medical Research, Clayton, VIC, Australia) using the automated Applied Biosystems 3130xl Genetic Analyzer and the Applied Biosystems BigDye Terminator 3.1 reaction kit (Applied Biosystems, Carlsbad, CA, USA). Sequence files were analysed using the software 4 Peaks (v1.7.1) (mekentosj.com) and Basic Local Alignment Tool (BLAST; NCBI; http://blast.ncbi.nlm.nih.gov) to confirm that the correct sequences were amplified. The sequences were further aligned using the ClustalW2 alignment software tool (EMBL, Germany) and UGENE^49^ multiple alignment tool to generate a phylogenetic tree.

### Generation of MM xenografts and bioluminescent imaging

2.5×10^5^ U266 cells were transplanted into NSG mice by tail vein injection. Mice were imaged using a Lumina III XR bioluminescent imaging system (Perkin Elmer, Waltham, MA, USA) starting at 4 weeks post-transplant. Briefly, mice were injected intraperitoneally with sufficient luciferin (Perkin Elmer) for a final concentration of 30 mg/kg. After 10 min, mice were anaesthetized by inhalation of isofluorane and placed in the instrument. To acquire and analyse bioluminescent data, Living Image software (Perkin Elmer) was used. Regions of interest were drawn to encompass the entire mouse and total flux data were recorded.

### Post-mortem FACS sorting of donor cells for analysis

Following final imaging at 11 weeks post-transplantation, NSG mice that received undepleted and 7-day depleted U266 cells were killed. Bone marrow from both femurs and tibiae was obtained by flushing with PBS. The spine was isolated from the mouse and marrow cells were obtained by crushing the spine in a 40 *μ*m cell strainer (Falcon, Fisher Biotec, Wembley, WA, Australia) using the rubber end of a 5-ml syringe plunger (Falcon, Fisher Biotec). Following red blood cell lysis, cells were stained with APC-conjugated anti-human CD138 (Miltenyi, Bergisch Gladbach, Germany). All GFP+ and/or CD138+ cells were collected by FACS sorting on a FACS Aria (BD). Collected cells were centrifuged, the supernatant was removed and the cell pellet was snap frozen for subsequent analyses.

### 143B xenografts

Female BALB/c Nude mice, 6–8 weeks of age, received right flank injections of 1x10^6^ cells/mouse in 200 *μ*l of 1 : 1 mixed cell suspension and Matrigel. Tumour size was measured using digital calipers and volumes were calculated according to the formula: tumour volume (mm^3^)=(width^2^×length)/2. Mouse body weight and tumour volumes were measured daily. Experiments were terminated once tumour size exceeded 800mm^3^ or in the absence of tumour development 5 months post-inoculation. At end point, mice were killed by cervical dislocation, tumours were excised using aseptic techniques and divided for histology, RNA and DNA extraction, or cell sorting.

### Histology

Tumours were fixed in 10% neutral-buffered formalin for 24 h and changed into 70% ethanol before processing into paraffin. Parrafin-embedded tumour sections were analysed by histology after generating 4 *μ*m sections and staining with haematoxylin and eosin.

### Isolation of tumour cells by flow cytometry

Established heterogeneous tumour cell lines were sorted according to viability, singlets and cell size. Briefly, the heterogeneous cell suspension was incubated in LIVE/DEAD Fixable Violet Dead Cell Stain Kit (Life Technologies; L34955) to distinguish viable and dead cells and analysed by flow cytometry using the Mo-Flo BTA analyzer. Following gating for viable cells and singlets, three populations of cells were identified based on cell size. All three populations were individually isolated for analysis.

### DNA and RNA extraction

Total DNA and RNA were extracted using the DNeasy Blood and Tissue Kit (Qiagen) and RNeasy Mini Kit (Qiagen), respectively, according to the manufacturer’s instructions. The DNA samples were treated with RNase A solution (Qiagen) and Proteinase K Solution (Qiagen) at 65 °C for 10 min and RNA samples were treated with DNase I (Qiagen) for 20 min at room temperature.

### Reverse transcriptase PCR

cDNA was synthesized, according to the manufacturer’s instructions, using the Superscript III First-Strand Synthesis System (Life Technologies). *β*-Actin was chosen as the housekeeping gene to normalize the expression results following the real-time PCR (Supplementary Table S9).

### Real-time PCR: mtDNA copy number and mRNA expression analysis

Real-time PCR (quantitative PCR, qPCR) was performed on purified total DNA using the Rotor-Gene 3000 (Corbett Research, Cambridge, UK). Two microliters of DNA template were mixed with 10 *μ*l of 2X SensiMix (Bioline), 1 *μ*l of each forward and reverse primers (Sigma) (Supplementary Table S9) at a concentration of 5 *μ*M and 6 *μ*l distilled water to a final volume of 20 *μ*l. The number of mtDNA copies/cells and relative expression levels of *POLGA* were quantified against external standards of *β*-globin and mtDNA, using real-time PCR, as previously described.^14^ mRNA expression levels were determined by the ΔΔCt method, as described.^14^

### Gene expression microarray analysis

Purified total RNA from both early and late progression tumours derived from each 143B cell line was labelled, amplified and hybridized to HumanHT-12 v4 Expression BeadChip Kit (Illumina, Scoresby, VIC, Australia) in triplicate though the Australian Genome Research Facility Ltd (AGRF; Walter Eliza Hall Institute of Medical Research, Melbourne, VIC, Australia), according to the manufacturer’s protocols. Expression data were analysed using GenomeStudio Gene Expression Module (Illumina) and the limma package of R Bio-conductor^50^ (http://www.r-project.org).

### Immunoprecipitation of methylated DNA (MeDIP) analysis

MeDIP was performed, as previously described,^14^ with minor modifications. Purified DNA was fragmented to sizes between 200–1000 bp using the S220 Focused-ultrasonicator (Covaris, Woburn, MA, USA) and the software SonoLab 7 (Covaris). Fragmented DNA was denatured at 95 °C for 10 min and cooled on ice for 5 min. Following denaturation, 3 *μ*g of single-stranded DNA fragments were incubated with 2 *μ*g of either anti-5-methylcytosine (5mC; Active Motif, Carlsbad, CA, USA) or anti-5-hydroxymethylcytosine (5hmC; Active Motif) antibodies with 20 *μ*l of Protein G Dynabeads (Invitrogen, Carlsbad, CA, USA) in 500 *μ*l of IP buffer, consisting of 10 mM sodium phosphate, pH 7.0, 140 mM sodium chloride and 0.05% (v/v) Triton X-100, at 4 °C overnight. Samples were washed three times with 700 *μ*l IP buffer after overnight incubation. Immunoprecipitated DNA was released from the beads and eluted in 250 *μ*l of Proteinase K digestion buffer, consisting of 5 mM Tris, 1 mM EDTA, pH8.0, 0.05% SDS with the addition of 7 *μ*l of Proteinase K (20 *μ*g/*μ*l; Qiagen) while rotating at 50 °C for 3 h. DNA samples were purified using phenol–chloroform and precipitated with 3 *μ*g of glycogen (Invitrogen) and 100% ethanol at −80 °C for a minimum of 2 h. DNA pellets were further washed with 70% ethanol and resuspended in distilled water.

### mtDNA next generation sequencing

Templates for next generation sequencing were generated by long PCR amplification of two overlapping fragments that cover the entire mitochondrial genome. The PCR reactions were prepared with 1× High Fidelity PCR buffer, 100 mM MgSO_4_, 1U of Platinum *Taq* High Fidelity (Invitrogen), 1 mM dNTPs (Bioline) and 10 *μ*M each of forward and reverse primers (Supplementary Table S9). PCR products were purified using the QIAquick PCR Purification Kit (Qiagen).

Purified amplicons from both long PCR reactions were combined at equal concentrations and used to generate amplicon libraries using the Ion Fragment Library kit and the Ion Express Template kit (Life Technologies), according to the manufacturer’s instructions. Libraries were loaded onto 318 chips for sequencing on an Ion Torrent Personal Genomic Machine (PGM). Sequences were then aligned to the reference sequence using CLC Genomic Workbench (v5.5.1). Variant selection was performed using CLC Genomic Workbench (v5.5.1), as previously described.^28^ For detection of variants, a minimum mutation threshold of 3% was applied prior to analysis.

### RNA sequencing

An Agilent Bioanalyser Nanochip (Agilent, Melbourne, VIC, Australia) was used for quality control of total RNA. cDNA libraries were synthesized and prepared using the Illumina Truseq Stranded mRNA Kit (Illumina), according to the manufacturer’s instructions. cDNA libraries were then sequenced using the HiSeq 2000 sequencing system (Illumina) at AGRF. The primary sequence data were generated using the Illumina CASAVA 1.8.2 pipeline. The sequence reads were aligned against the Homo sapiens genome (Build version HG19) and mapped using the Tophat aligner (v1.3.1; http://ccb.jhu.edu/software/tophat/manual.shtml). The transcripts were assembled and tested for differential expression using the Cufflinks tool (v2.2.1; http://cole-trapnell-lab.github.io/cufflinks/).

### Computational pathway analysis

Normalized expression data generated from both microarray and RNA-seq were uploaded into Ingenuity Pathway Analysis software (IPA; Qiagen). Expression data from the microarray were analysed with cutoffs of a fold-change in expression over 2 and *P*<0.05. The same cutoffs were applied to the RNA-seq data with the addition of the false discovery rate (*q*-value) <0.05. The selected genes were then mapped against the inbuilt KEGG pathways for selection of the most affected canonical pathways and networks.

### Statistics analysis

Graphical output and statistical analyses were performed using the Prism 5 software package version 5.0d (GraphPad Software, San Deigo, CA, USA). One-way ANOVA was used followed by Bonferroni *post hoc* tests. Significance was considered when *P*<0.05. All data are represented as mean±S.E.M.

## Data deposition

Mitochondrial genome sequence data for 143B^100^, 143B^NSC^ and 143B^GBM^ cells have been deposited in the GenBank nucleotide core database under the accession codes KT946592, KT946593 and KT946594. Next generation sequencing data for the mitochondrial genomes of all 143B and U266 samples have been deposited in the NCBI Sequence Read Archive (SRA) under the accession code PRJNA29 5347. RNA sequencing data for all U266 and HSR-GBM1 samples have been deposited in the NCBI Sequence Read Archive (SRA) under the accession code PRJNA296542. Illumina gene expression microarray data for all 143B samples have been deposited in the Gene Expression Omnibus (GEO) database under the accession code GSE73120.

## Notes

Supplementary Extended Data Figure 1

Supplementary Extended Data Figure 2

Supplementary Extended Data Figure 3

Supplementary Extended Data Table 1

Supplementary Extended Data Table 2

Supplementary Extended Data Table 3

Supplementary Extended Data Table 4

Supplementary Extended Data Table 5

Supplementary Extended Data Table 6

Supplementary Extended Data Table 7
