# Supplementary figures and images for: Mitochondrial DNA plasticity is an essential inducer of tumorigenesis
Source: Cell Death Discov. 2016 Apr 4;2:16016–. doi: 10.1038/cddiscovery.2016.16 (PMC4979526; doi:10.1038/cddiscovery.2016.16)

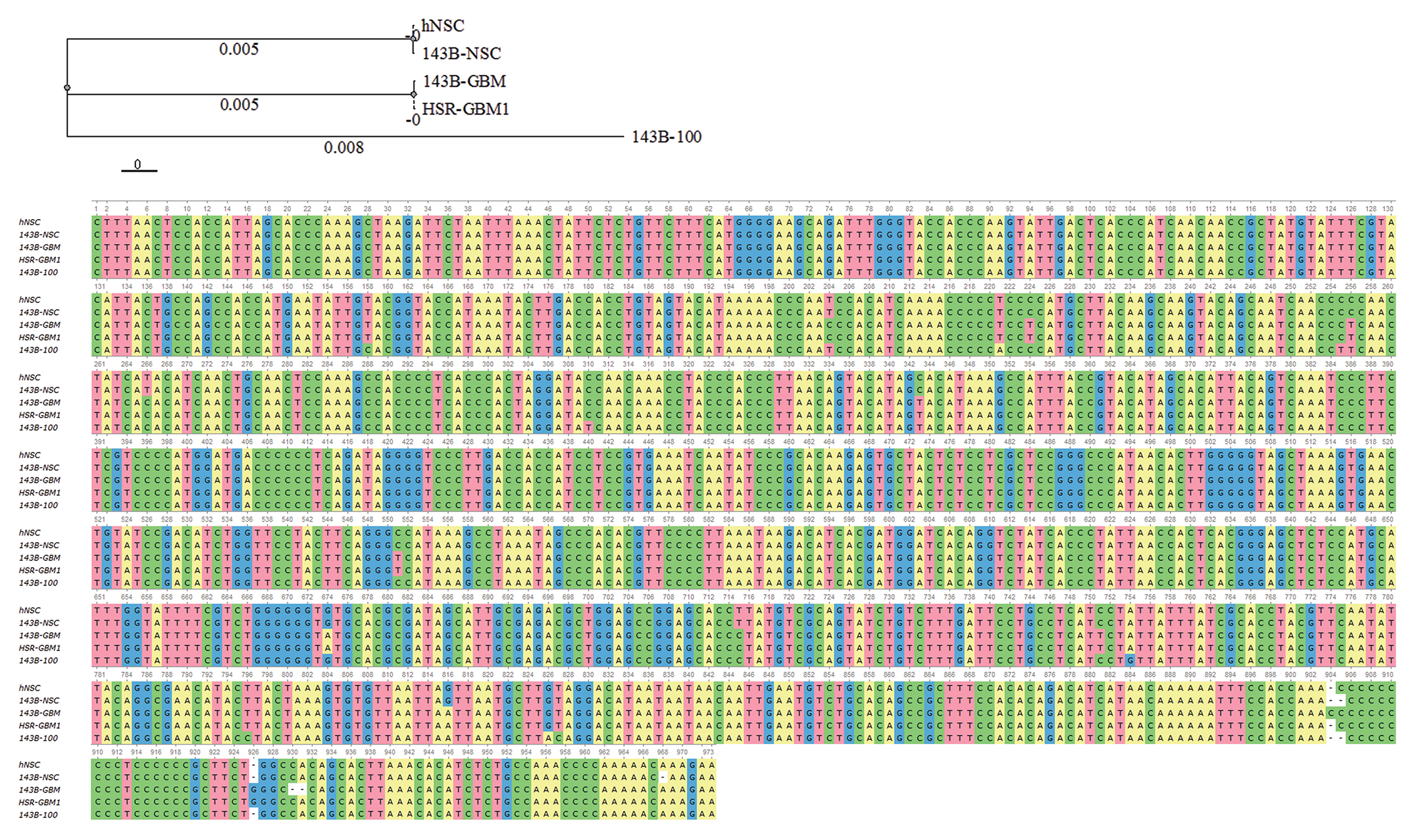

Supplement: Supplementary Extended Data Figure 1 [file cddiscovery201616-s1.tiff]

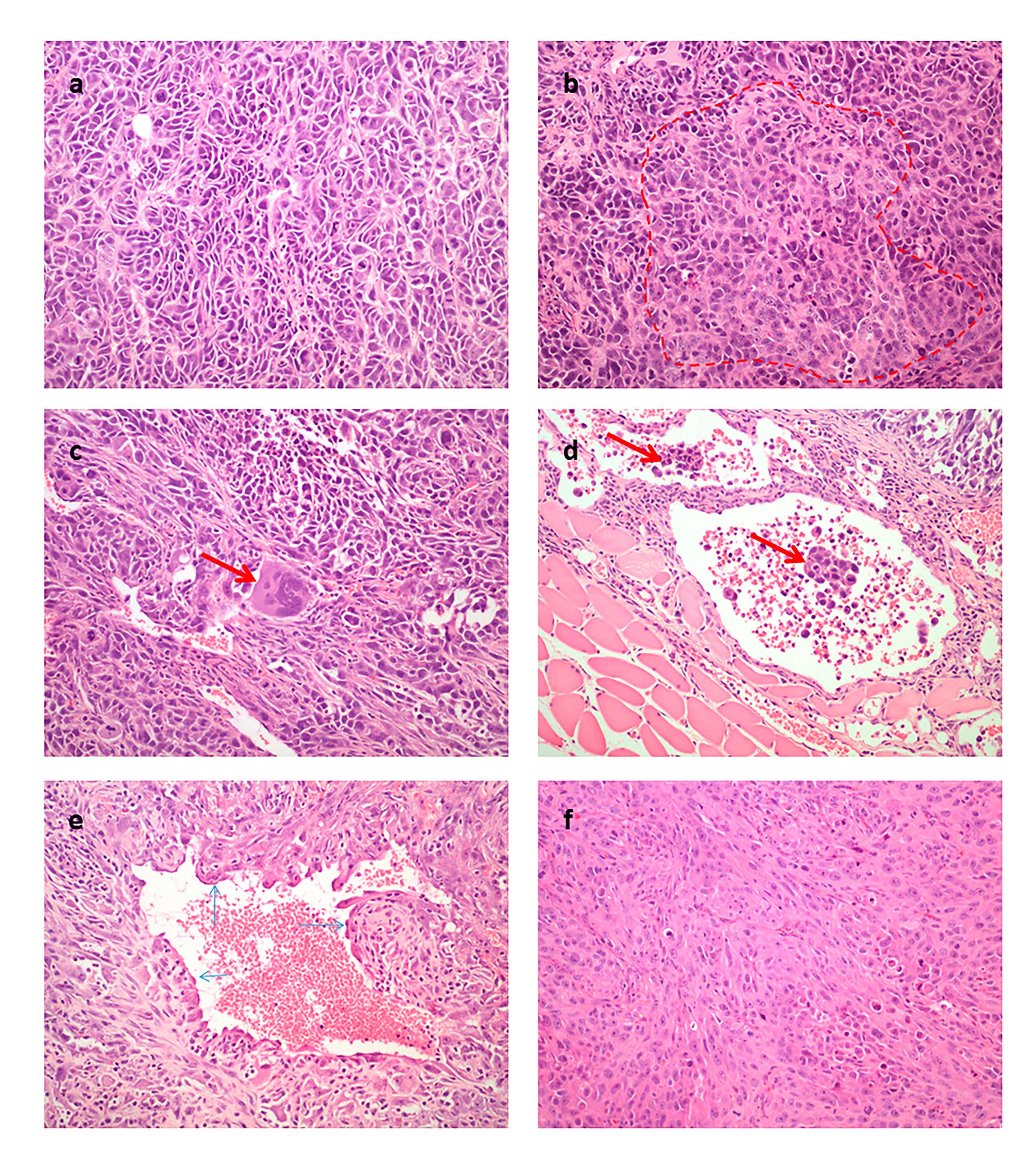

Supplement: Supplementary Extended Data Figure 2 [file cddiscovery201616-s2.tiff]

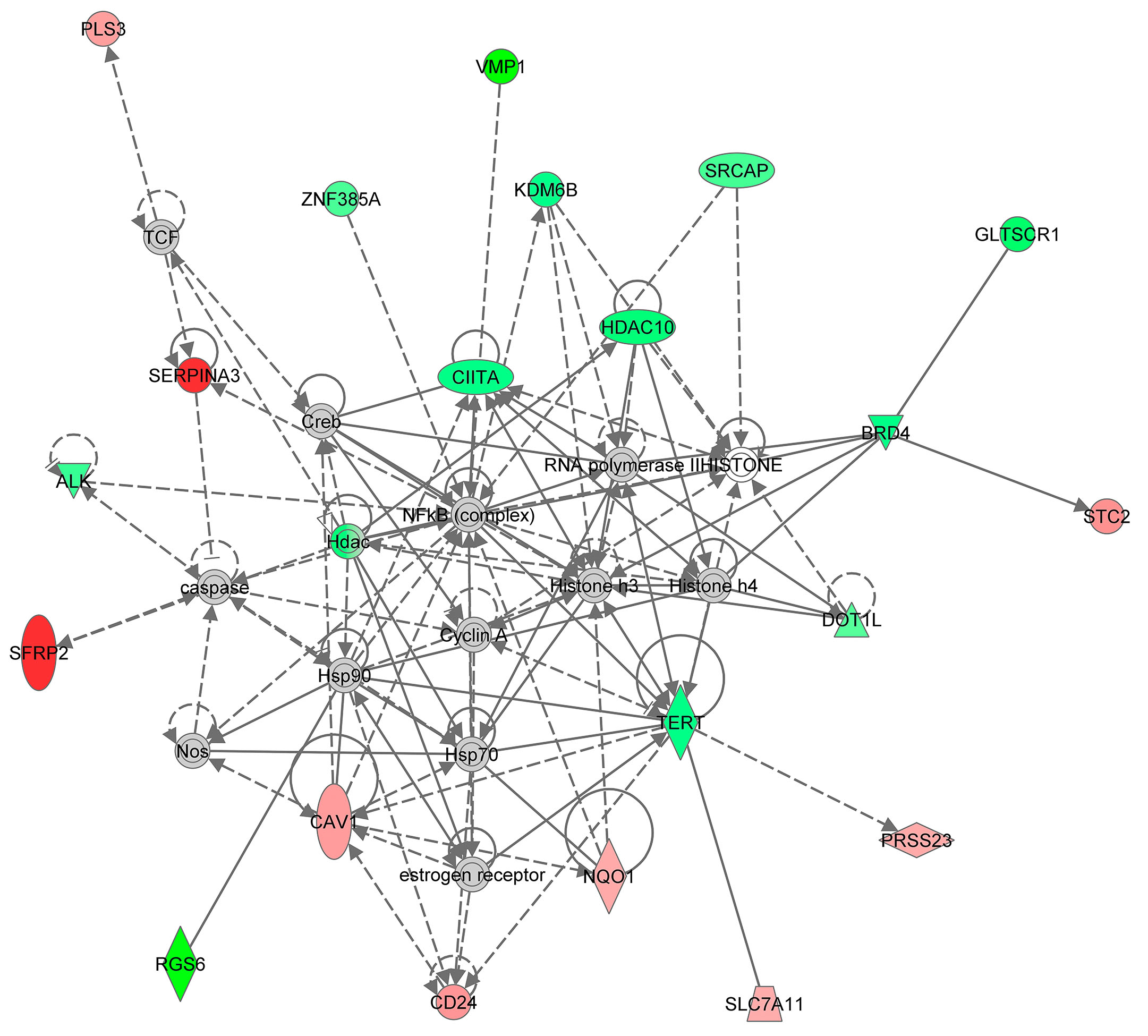

Supplement: Supplementary Extended Data Figure 3 [file cddiscovery201616-s3.jpg]

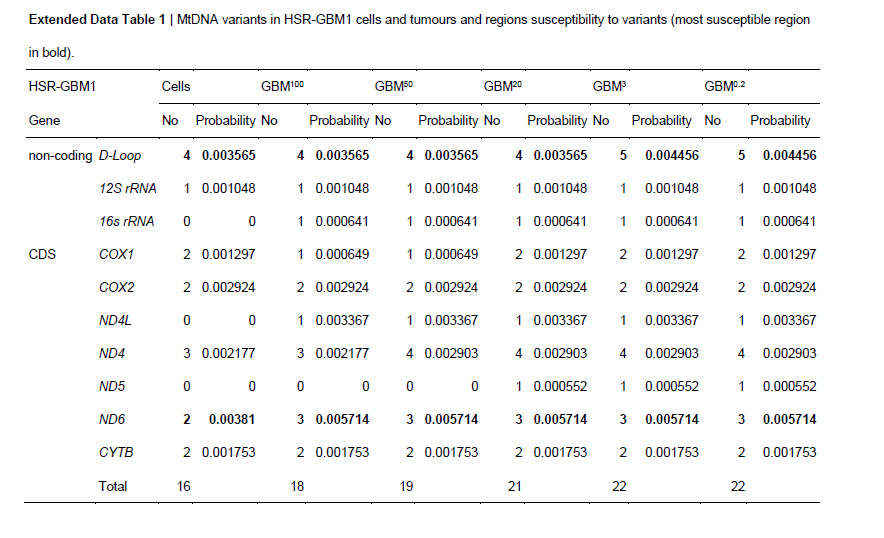

Supplement: Supplementary Extended Data Table 1 [file cddiscovery201616-s4.jpg]

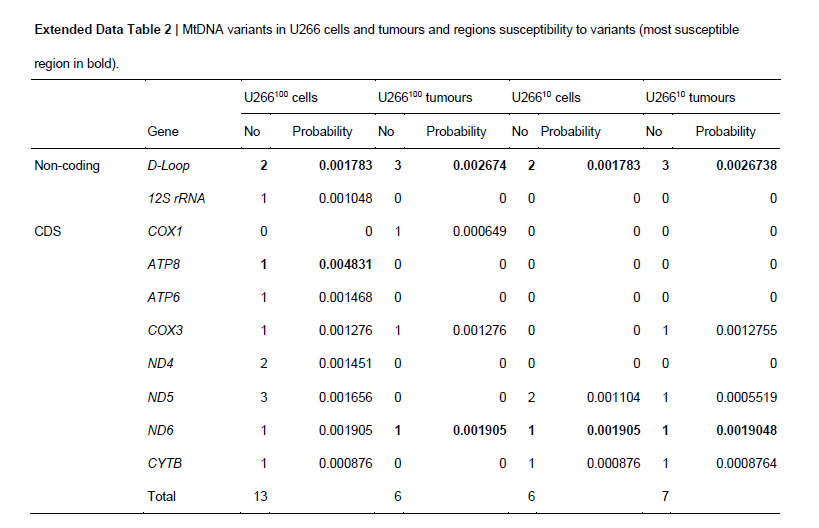

Supplement: Supplementary Extended Data Table 2 [file cddiscovery201616-s5.jpg]

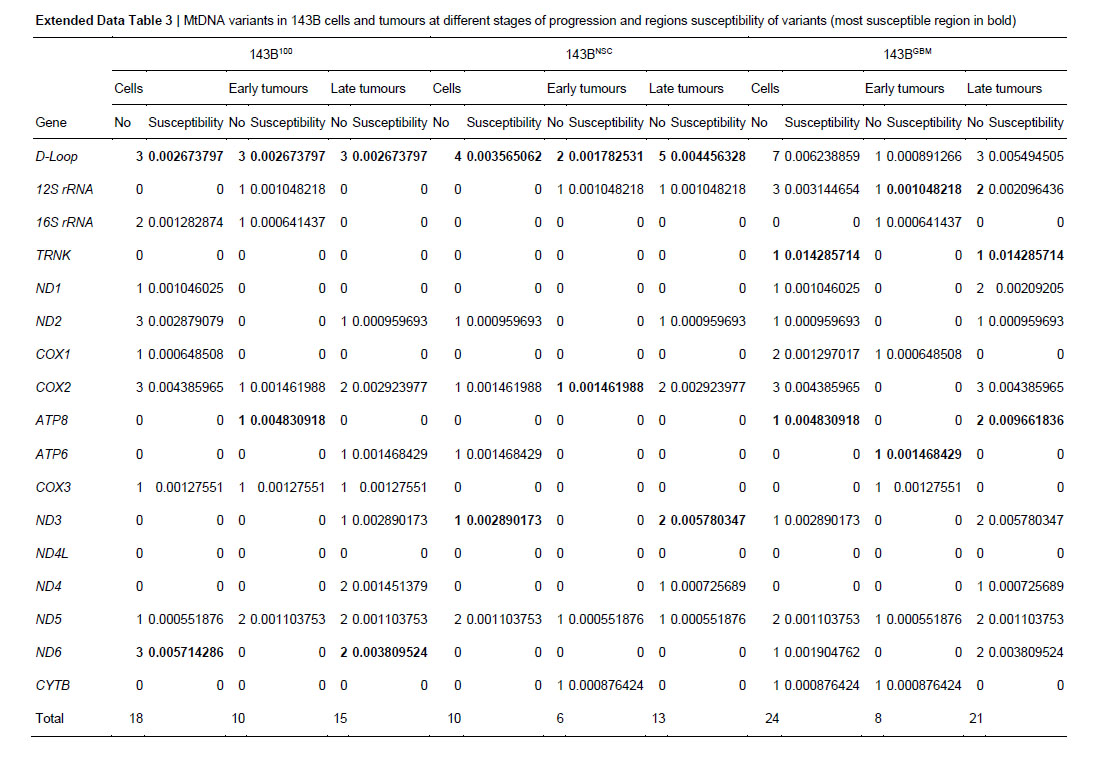

Supplement: Supplementary Extended Data Table 3 [file cddiscovery201616-s6.jpg]

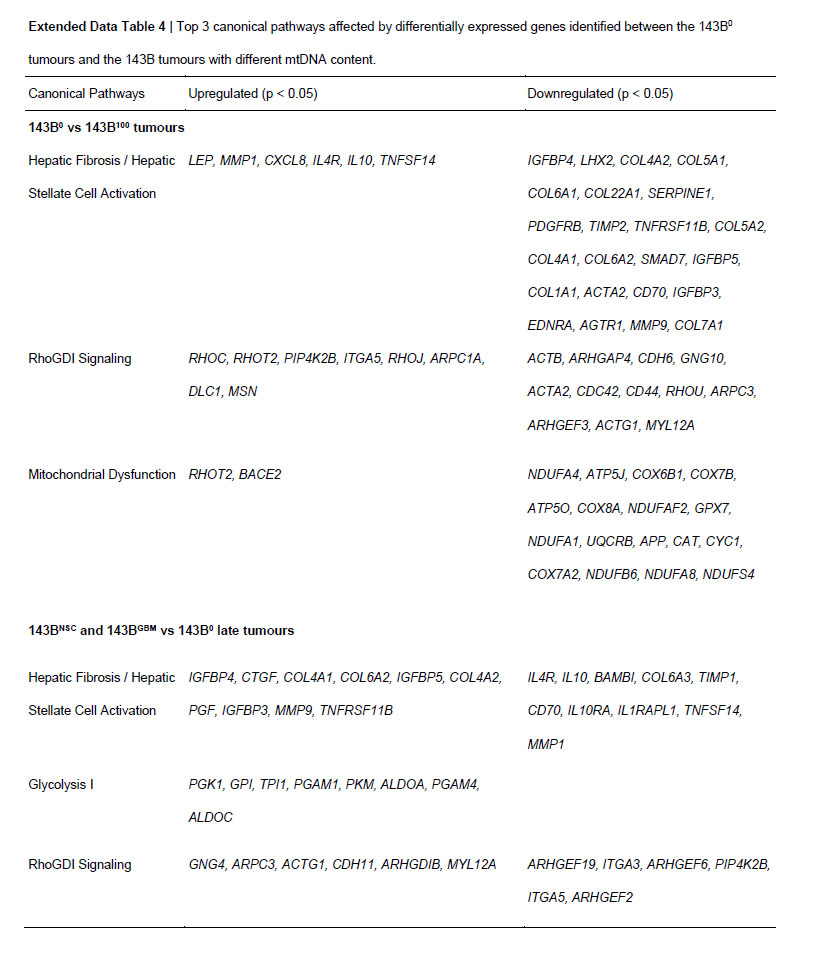

Supplement: Supplementary Extended Data Table 4 [file cddiscovery201616-s7.jpg]

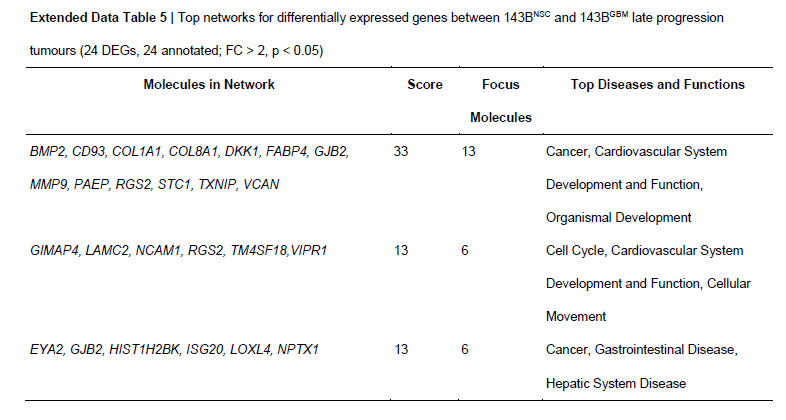

Supplement: Supplementary Extended Data Table 5 [file cddiscovery201616-s8.jpg]

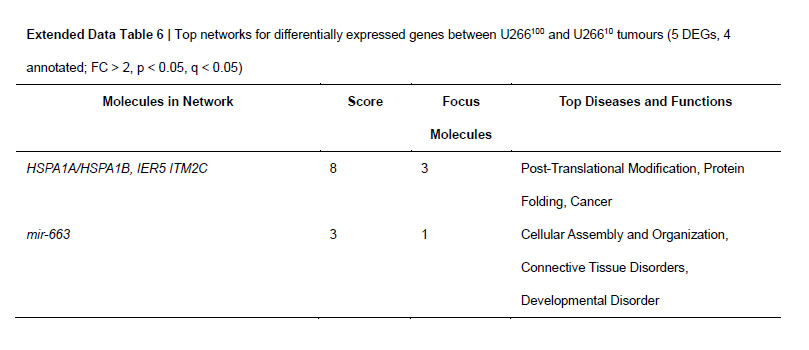

Supplement: Supplementary Extended Data Table 6 [file cddiscovery201616-s9.jpg]

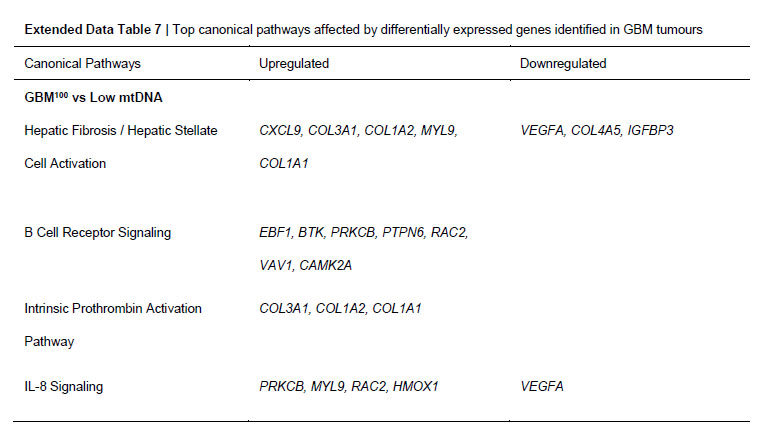

Supplement: Supplementary Extended Data Table 7 [file cddiscovery201616-s10.jpg]
