# Supplementary material for: Mitochondrial DNA plasticity is an essential inducer of tumorigenesis
Source: Cell Death Discov. 2016 Apr 4;2:16016–. doi: 10.1038/cddiscovery.2016.16 (PMC4979526; doi:10.1038/cddiscovery.2016.16)
Supplement: Supplementary Tables [file cddiscovery201616-s11.pdf]

Supplementary Table 1 | Top networks for differentially expressed genes between $143B^{100}$ and $143B^{0}$ late progression tumours (951 DEGs, 280 annotated; FC > 2, $p < 0.05$)

Molecules in Network	Score	Focus Molecules	Top Diseases and Functions
ANKRD13D, APP,BACE2, BRSK1, CCDC106, CLIC6, CMTM3, DDX55, ENY2, FAM127A, FAM127B, FAM64A, GPM6B, GPT2, HAGH, HCFC1R1, LARP6, MRPL20, MRPS24, OLFML2A, PDDC1, PLEKHO2, PNPO, PRR14, RTN1, RUFY3, SCO2, SERF1A/SERF1B, SPC24, ST3GAL6, TMEM175, TSPAN13, UGGT2, WARS2, ZNF483	48	35	Nervous System Development and Function, Drug Metabolism, Molecular Transport
ANXA2, ATP5J, EIF4E2, FBXO18, HNRNPH2, HNRNPK, HNRNPUL1, LIMCH1, MAGOH, MMP1, PABPC1, PPFIBP1, RBM3, RBM25, RPA3, RPL6, RPL14, RPL17, RPL21, RPL29, RPL26L1, RPL36A, RPL7L1, RPLP0, RPLP1, S100A4, SPTBN1, TAGLN2, TIMP3, TSR1, ZNF598	43	31	RNA Post-Transcriptional Modification, Infectious Disease, Hematological Disease
BCL2L13, BIRC3, CCPG1, DDX47, DLGAP5, DRG1, FAM175A, FAM50B, GJA1, GLS, HIST2H2AA3/HIST2H2AA4, HMGN4, LMNA, MBD4, MGST3, MUM1L1, NUP155, PGM1, PHF5A, PNP, RBM12, RBM33, SLTM, SPANXC (includes others), SUMO2, SUZ12, TDP1, TERF2IP, TOMM34, USP13	41	30	Cellular Assembly and Organization, Cellular Function and Maintenance, Connective Tissue Development and Function
ABR, AKAP8L, ARFGAP1, ARHGEF3, BASP1, CADM1, CPSF2, DGKD, DOCK6, ELOVL5, ELOVL6, GRB14, HNRNPL, KDELR3, KHDRBS3, LAMTOR2, LARS, MAN2C1, PLEKHA2, PPP6R2, PTP4A2, RABGGTB, RRAGA, RSU1, SYMPK, TNFRSF14, TNFSF14, TPM1, TRIB3	39	29	Infectious Disease, Cellular Assembly and Organization, Cellular Function and Maintenance
ANXA1, ATG7, ATG4B, FOXK1, GBAS, HIST2H2AC, HMGCS1, IER3, IGSF3, LONP1, MAP1LC3A, MTF2, MYO10, NFKBIA, PFAS, PGK1, PLD3, PSMC1, PSMC4, PSMD6, PSMD14, PSME4, RBBP7, SPOCK1, TCF20, UBAP1, UBB	34	27	Cellular Assembly and Organization, Infectious Disease, Cellular Response to Therapeutics
ALDH1A3, AP4B1, BAG4, BARX1, CARD8, DNTTIP1, FAM46A, FOXD1, FUCA1, LAMP2, LDB2, MID1, MLKL, MLLT6, NGFRAP1, PDLIM1, PNPT1, SLC7A5, STK36, TNFRSF25, UBE2C, UBE2E2, UBE2E3, UBE2G2, ZFAND5	32	26	Embryonic Development, Nervous System Development and Function, Organ Development
ANGPTL2, CCT6A, CDH6, CYGB, DCTN4, DYNC1H1, DYNC1LI2, DYNLL1, DYNLT1, DYNLT3, ESM1, FAM107B, LPHN2, MELK, NDEL1, NT5E, PLXNA2, PRC1, PRSS3, SHISA2, STMN2, STRIP2, TUBA1A, TUBB2A, TUBB2B, TUBB4B	31	26	Cancer, Connective Tissue Disorders, Skeletal and Muscular Disorders

ARHGAP22, BHLHE40, CBX3, CCDC86, CEBPB, DPY30, EPAS1, ETFB, ETV5, GCHFR, H3F3A/H3F3B, HMGB2, HSD17B7, KCNF1, KLHDC4, LEP, LMO4, MAT2A, NFKBIZ, OGT, PLAT, SERPINF1, SLC9A8, SP3, SPRY1, TNFRSF11B	31	26	Embryonic Development, Organ Development, Organismal Development
AKAP12, CH25H, CHGB, CPE, CSNK1G1, FABP5, FLNC, ICA1, ISG20, ITPR3, JOSD1, MKKS, MT1H, P4HA2, PDLIM3, PLEKHG4, PPP2R5C, RPS6KA2, SEC61A1, SEC61B, SEC61G, SLC2A1, STK24, STUB1, UTP14A	30	25	Cell Cycle, Infectious Disease, Carbohydrate Metabolism
ALG5, ANPEP, CSDE1, EEF1A2, EIF2B5, FXYD6, HAUS2, HIST1H4C, MCOLN1, MINOS1-NBL1/NBL1, MRPL22, MRPS21, NDRG1, PDCD4, PPP1R15A, RPS15, RPS24, RPS26, RPS15A, SCG2, SERPINE2, SLC35E1, STMN3, TMEM126A, UBQLN1	30	25	Protein Synthesis, Developmental Disorder, Hematological Disease

Supplementary Table 2 | Top networks for differentially expressed genes between 143B cybrids (143B^{NSC} and 143B^{GBM}) and 143B^{0} late progression tumours (379 DEGs, 203 annotated; FC > 2, p < 0.05)

Molecules in Network	Score	Focus Molecules	Top Diseases and Functions
ADM, AKAP12, AKR1C4, ARL6IP5, ATG7, ATP9A, BNIP3L, CCDC86, DBI, FILIP1L, FLNC, GJA1, HSD17B7, KLF2, LDHA, NDRG1, P4HA1, P4HA2, PFKFB3, PGK1, PKM, PLD3, PLOD2, PRDX5, SLC2A1, UPP1	42	26	Carbohydrate Metabolism, Amino Acid Metabolism, Post-Translational Modification
ACTG1, AIRE, CDKN1A, CEBPB, DLGAP5, DMAP1, ERCC1, HAUS2, HJURP, ICA1, IFITM2, IFITM3, IL10, IL10RA, KIF20A, LSM3, RNU1-1, RNU1-3, RNU1-4, RNVU1-18, SNAPC1, SSBP1, TAP1, TM4SF18	37	24	Cellular Development, Hematological System Development and Function, Hematopoiesis
AGR2, CADM1, CDH11, COL4A1, COL4A2, COL6A2, COL6A3, CSF2RA, DENR, GRB14, HEY1, HOXB8, JAG1, MFAP2, MGLL, MMP1, NRP1, PTP4A2, RRBP1, SPARC, TAGLN, THBS1, TMEM45A	36	23	Connective Tissue Disorders, Cardiovascular System Development and Function, Cellular Development
AGPAT9, ALDOA, ALDOC, ASNS, C1QBP, CD93, CHRNA1, DAPP1, DDIT4, EIF2AK4, FABP5, FUT8, GABRB1, GBE1, KIFC2, MTHFD2, OSBPL10, PCK2, PITX1, PRDM1, SERPINI1, SQLE, TRIB3	36	23	Carbohydrate Metabolism, Small Molecule Biochemistry, Respiratory Disease
AKR1C3, ALDH1A3, CARD8, DNTTIP1, E2F7, ENY2, FASN, FDFT1, GLRX, INSIG1, KAT2A, LDB2, NGFRAP1, NRIP1, PNPT1, RIPK4, RXRB, SCD, SLC16A3, SLC7A5, T3-TR-RXR	29	20	Organismal Development, Lipid Metabolism, Small Molecule Biochemistry
AARS, ABLIM1, ADA, AMY2A, ANGPT2, ARHGDIB, CALD1, CD68, CTSB, CTSL, FKBP1A, HOMER3, IFI44, ITPR3, LIME1, NDP, OSCAR, PMAIP1, PRSS3, SPOCD1	25	20	Inflammatory Disease, Infectious Disease, Developmental Disorder
ACAT2, AHR, FABP4, GPI, HERC5, LIPA, MLPH, PPP6R2, RASSF2, RBCK1, SDCBP, SLC25A51, TMEM126A, TNK2, TYMS, ULK1, USP49, WDR41	24	18	Digestive System Development and Function, Hepatic System Development and Function, Organ Morphology
ATP5O, ATP6AP1, BTF3, CBX6, CDK5RAP2, CHST15, GOLGA8A/GOLGA8B, HIST1H1C, KPNA3, LARP6, NTSR1, PGAM1, PGAM4, RPL21, STK36, SULT1A1, TPI1	24	17	Cell-To-Cell Signaling and Interaction, Drug Metabolism, Small Molecule Biochemistry
BHLHE40, CD70, CHN1, DACH1, EPHA4, GRB10, IGFBP3, IGFBP4, IGFBP5, KLC1, MMP3, NOX4, PDCD4, PGF, PRSS2, TFPI2	22	16	Organ Morphology, Skeletal and Muscular System Development and Function, Tissue Morphology

ADD3, APOE, BNIP3, CHD5, CKAP4, CTGF, ETS1, GREM1, IL1RAPL1, LOX, MMP9, S100A2, SERPINE2, SLCO4A1, SORCS3, SPP1	22	16	Cardiovascular System Development and Function, Cellular Movement, Cardiovascular Disease

Supplementary Table 3 | Top 3 canonical pathways affected by differentially expressed genes identified between the $143B^{NSC}$ tumours and the $143B^{GBM}$ tumours

Canonical Pathways	Upregulated ($p < 0.05$)	Downregulated ($p < 0.05$)
**$143B^{NSC}$ vs $143B^{GBM}$ tumours**		
Adipogenesis pathway	BMP2, FABP4	TXNIP
Hepatic Fibrosis / Hepatic Stellate Cell Activation	COL1A1, COL8A1, MMP9	
Role of Osteoblasts, Osteoclasts and Chondrocytes in Rheumatoid Arthritis	COL1A1, BMP2, DKK1	

Supplementary Table 4 | Top networks for differentially expressed genes between early and late progression 143B^{NSC} tumours (33 DEGs, 27 annotated; FC > 2, p < 0.05)

Molecules in Network	Score	Focus Molecules	Top Diseases and Functions
CDC42SE1, CLEC2D, CUEDC1, GAS1, GOLIM4, GYPC, HES4, HOXB2, HOXB5, MT1A, RNU1-1, RNU1-3, RNU1-4, RNVU1-18	35	14	Connective Tissue Development and Function, Embryonic Development, Nervous System Development and Function
CDC42SE1, CHRNA1, COL1A1, DDIT4, HOXB5, IGFBP5, KIT, MT2A, RPL9, RPL21, RPL10A, SULF2, TIMP3, TNFRSF11B	35	14	Cellular Function and Maintenance, Connective Tissue Development and Function, Skeletal and Muscular System Development and Function
SNORD3A	3	1	Gene Expression, Infectious Disease, Organismal Development

Supplementary Table 5 | Top networks for differentially expressed genes between early and late progression $143B^{GBM}$ tumours (30 DEGs, 16 annotated; FC > 2, $p < 0.05$)

Molecules in Network	Score	Focus Molecules	Top Diseases and Functions
CLEC2D, DRAP1, GOLIM4, IFI27, NPTX1, RPL21, RPL10A, SIGLEC12	20	8	Cellular Development, Cellular Growth and Proliferation, Cell Cycle
ACKR3, APOE, DNAJB1, HSPA6, HSPA1A/HSPA1B, ISG15, PPAP2C, S100A4	20	8	Developmental Disorder, Hereditary Disorder, Metabolic Disease

Supplementary Table 6 | Top networks for differentially expressed genes between U266^{100} and U266^{10} cells pre-inoculation (16 DEGs, 14 annotated; FC > 2, $p < 0.05$, $q < 0.05$)

Molecules in Network	Score	Focus Molecules	Top Diseases and Functions
IFIT1, IFIT5, ISG15, mir-573, mir-663, mir-1204, MX1, NR4A2, PHC2, PYROXD2	26	10	Cellular Development, Hematological System Development and Function, Hematopoiesis
COL17A1, PCSK1N, TMEM160	6	3	Connective Tissue Disorders, Dermatological Diseases and Conditions, Developmental Disorder
ANKRD30BL	3	1	Dermatological Diseases and Conditions, Developmental Disorder, Hereditary Disorder

Supplementary Table 7 | Top networks for differentially expressed genes between GBM^{100} and extensively depleted GBM (GBM^{3} and $GBM^{0.2}$) tumours (101 DEGs, 94 annotated; FC > 2, p < 0.05, q < 0.05)

Molecules in Network	Score	Focus Molecules	Top Diseases and Functions
AMH, ANXA1, CAMK2A, CD24, CORO1A, DTX1, EBF1, EMP1, FMNL1, HCLS1, KCNK5, MAN1A1, MYL9, PALM, PRKCB, RGS6, SATB1, SMIM3, TRPM3, UGT8	40	20	Cellular Development, Cellular Growth and Proliferation, Hematological System Development and Function
APOE, BTK, COL1A1, COL1A2, COL3A1, COL4A5, DLK1, DLL3, Fibrinogen, *FLI1, HMOX1, IDO1, IGFBP3, INSM1, KCNN4, LCP1, NDRG1, SLC22A17, THBS4, VEGFA*	38	19	Cancer, Gastrointestinal Disease, Organismal Injury and Abnormalities
AMOT, ARID1A, BIN1, DSCAML1, FNBP1L, H2AFJ, MYO1G, NCKAP1L, NEK9, PLEK, POTEE/POTEF, SLC38A5, SNORA8, SYNE1, TMEM57	28	15	Cancer, Organismal Injury and Abnormalities, Reproductive System Disease
CXCL9, CXCL10, DOCK2, GADD45G, IRF8, LBH, PTGS1, PTPN6, RAC2, RASGRP3, SLC12A7, TMEFF2, VAV1	21	13	Cellular Function and Maintenance, Cellular Movement, Hematological System Development and Function
ABCA8, CACNG8, HOXB3, IKZF1, KCNE5, PLEKHH2, RUNDC3A, SASH3, STOX2, VSTM2A	16	10	Cell Morphology, Humoral Immune Response, Lymphoid Tissue Structure and Development
EIF4A2, FOXP1, PCSK1N, PKIB, RORB, RTP5, SHD, STAC2, TTC9	15	9	Organ Morphology, Reproductive System Development and Function, Embryonic Development
CIT, ENPP2, let-7, MYO5B, RARRES2, RPL23A, SLC12A7, VMP1	12	8	Cellular Compromise, Cellular Movement, Hematological System Development and Function

Supplementary Table 8 | Top networks for differentially expressed genes between GBM[100] and GBM[50] late progression tumours (136 DEGs, 125 annotated; FC > 2, p < 0.05, q < 0.05)

Molecules in Network	Score	Focus Molecules	Top Diseases and Functions
ALK, BRD4, CAV1, CD24, CIITA, DOT1L, GLTSCR1, HDAC10, KDM6B, NQO1, PLS3, PRSS23, RGS6, SERPINA3, SFRP2, SLC7A11, SRCAP, STC2, TERT, VMP1, ZNF385A	40	21	Cancer, Cell Cycle, Cellular Development
ACTG2, ADAMTS12, AMOT, ATP1B2, CR1, CTNNA2, DDX25, DES, GEM, LYZ, MSTN, MYH7, MYL9, MYLK, MYO6, MYOG, SLC1A1, SYNGAP1, TNNT2	35	20	Skeletal and Muscular System Development and Function, Skeletal and Muscular Disorders, Cardiovascular System Development and Function
ACTA2, COL12A1, COL1A1, COL1A2, COL3A1, COL5A2, DAPK1, FBN1, GUCY1B3, LOX, POSTN, PROM1, PROS1, PRSS35, SERPING1, SLC12A7, TGFBI, THBS4	34	18	Connective Tissue Disorders, Cellular Assembly and Organization, Cellular Function and Maintenance
AQP1, CD38, COLEC12, CPSF1, DUOX1, HMOX1, HSPB6, KDR, NFIC, NLGN2, PPARD, SFRP4, TUB, VASH2	24	14	Connective Tissue Development and Function, Skeletal and Muscular System Development and Function, Cancer
ABCA8, ATP8A1, COL14A1, CPNE7, CSRNP3, FAM65A, L3MBTL1, MAGEH1, NXPH4, PLXDC2, PRR12, TMEM45A, ZDHHC2	22	13	Lipid Metabolism, Molecular Transport, Small Molecule Biochemistry
ANKRD54, CACNG8, FBN3, NEAT1, NFIC, PGAM2, PROSER3, RHCG, SLC17A8, USB1, VMP1	18	11	Cardiovascular Disease, Behavior, Nervous System Development and Function
CACNG5, DTX4, EPDR1, HOXB3, MLXIP, NFIC, RGS6, SLC9A3, SSPN, TRPM3, ZNF703	17	11	Dermatological Diseases and Conditions, Cellular Growth and Proliferation, Hematopoiesis
ATN1, BAI1, GPNMB, GPR135, KAL1, LENG8, NELFA, PTPN23, RPL31, SYNE1	15	10	Cell-To-Cell Signaling and Interaction, Cellular Growth and Proliferation, Nervous System Development and Function
DUOX1, ELF4, HSPB6, IRX1, PLXNB3, POSTN, RPL23A	10	7	Organismal Injury and Abnormalities, Carbohydrate Metabolism, Renal Degeneration

Supplementary Table 9 | Primers for PCR and qPCR

target region	primers	sequence
**mtDNA copy number**		
mouse ActB	F	AGCCTTGTCCCGGATGGAGA
	R	GCGCGACCAATCGGACTCAA
mouse mtDNA	F	CAGTCTAATGCTTACTCAGC
	R	GGGCAGTTACGATAACATTG
human Beta-Globin	F	CAACTTCATCCACGTTCACC
	R	GAAGAGCCAAGGACAGGTAC
human mtDNA	F	CGAAAGGACAAGAGAAATAAGG
	R	CTGTAAAGTTTTAAGTTTTATGCG
**MeDIP**		
Polg Exon2	F	CAGACCTCCACGTCGAACAC
	R	GACAACCTGGACCAGCACTT
**mRNA expression**		
CLEC2D	F	TTCCTATCCTGGGAGCAGGA
	R	GACATGTATATCTGATTTGGAACAA
RPL21	F	GGAATGCCCCACAAGGGTTA
	R	TCCAGGAAGCTATCTCGGCT
RPL10A	F	TATGATGCGTTTTTGGCCTCA
	R	TTTGGCCACCATGTTTTCGT
GOLIM4	F	AAGATGTTCTGCTTGCCCGA
	R	AGTGGAACGCAGAGAACCTG
ACTB	F	CAAAACCTAACTTGCGCAGA
	R	TTTTAGGATGGCAAGGGACT
**Long PCR for Ion Torrent Sequence**		
Long1	F	GACGGGCTCACATCACCCCATAA
	R	GCGTACGGCCAGGGCTATTGGT
Long2	F	GCCACAACTAACCTCCTCGGACTCCT
	R	GGTGGCTGGCACGAAATTGACC
